# Stone Cutting of the Ancient Greeks

**Published:** 1884-12

**Authors:** 


### STONE CUTTING OF THE ANCIENT GREEKS.

A RATHER interesting observation has recently been made upon the methods of st ne-cutting employed by the ancient Greeks. The marble blocks of which the Grecian masonry was composed are put together without mortar, and so nicely fitted that in many instances two adjacent stones have, as it were, grown together by the cohesion of their particles, brought into almost absolute contact, fracture made by a blow upon one passing directly into the other, just as if the two formed a single block. With regard to the fitting of the drums of columns, Mr. Penrose, the most scientific and practical of all investigators of Greek architecture, believes that the desired effect of close fitting was obtained by inserting a wooden pin as a pivot in the drill-holes which are always found in the centers of drums, and revolving each drum upon the one below it, first placing sand between the stones, until a perfect joint was obtained, in the same manner that glass stoppers are ground into bottles, and pieces of metal work of certain kinds fitted to each other. This explanation, which is probably the true one, solves the problem completely so far as the drums of columns are concerned, but throws no light upon the fitting of the other stones of the Grecian buildings, such as the blocks of the entablature, which are found to have joints as close as those of the columns, the edges of each block for a certain distance back from the face being polished, while the rest of the joint is slightly sunk, in order to allow the polished portions to be brought into perfect contact. As no sign of a pivot can be discovered on the stones, even if it were possible to revolve them in contact with each other, it is plain that a different process must have been used for fitting them, and an inscription discovered a few years ago gives us some idea of what the process may have been.

This inscription, which seems to have been a sort of official document, answering the purpose which would now be fulfilled by a printed specification, describes the construction of a temple, and stipulates particularly that the joints of every block of marble must be polished with a mixtuie of oil and vermilion. As vermilion, if the word so translated really refers to the pigment now known under that name, has no polishing quality, it has been suggested that the color was used simply to spread over the joints before trying the stones together. If any inequality existed in the surface of either stone, it would be immediately shown, on separating the stones after a momentary contact, by the transfer of color from one to the other, and the protuberant portion thus detected could then be rubbed down by hand to a uniform plane with the rest of the surface. A powder of red chalk is often used by marble-cutters for a similar purpose, and is quite possible that this may have been the only use of the vermilion paint, but there is some difficulty in accounting on this theory with the mixing of oil with the paint, which if used dry, would be quite as useful for its supposed purpose, and would be much more easily cleaned off the stone. There is no serious improbability in the supposition that the authors of the inscription may have confounded the true vermilion with the red oxide of iron, or crocus, which is a very efficient polishing agent, and if mixed with oil, and applied to the surface of a piece of marble, would serve admirably both to show where that sruface has been brought to coincide with a test plane and to reduce the inequalities which might, on trial, be found to exist.—*Manufacturer and Builder.*

